# Matrix Metalloproteinase-9 as an Important Contributor to the Pathophysiology of Depression

**DOI:** 10.3389/fneur.2022.861843

**Published:** 2022-03-18

**Authors:** Hongmin Li, Zhaofu Sheng, Suliman Khan, Ruiyi Zhang, Yang Liu, Yan Zhang, V. Wee Yong, Mengzhou Xue

**Affiliations:** ^1^The Department of Cerebrovascular Diseases, The Second Affiliated Hospital of Zhengzhou University, Zhengzhou, China; ^2^The Henan Medical Key Laboratory of Translational Cerebrovascular Diseases, Zhengzhou, China; ^3^Hotchkiss Brain Institute, University of Calgary, Calgary, AB, Canada; ^4^Department of Clinical Neurosciences, University of Calgary, Calgary, AB, Canada

**Keywords:** depression, matrix metalloproteinase 9 (MMP-9), neuroplasticity, minocycline, contributor

## Abstract

Matrix metalloproteinases (MMPs) are physiologically expressed in the central nervous system in neurons, astrocytes and microglia, and their aberrant elevation contributes to a number of diseases. Amongst the MMP members, MMP−9 has generated considerable attention because of its possible involvement in inflammatory responses, blood-brain barrier permeability, the regulation of perineuronal nets, demyelination, and synaptic long-term potentiation. Emerging evidence indicate an association between MMP−9 and the syndrome of depression. This review provides an updated and comprehensive summary of the probable roles of MMP−9 in depression with an emphasis on the mechanisms and potential of MMP−9 as a biomarker of depression.

## Introduction

The MMPs constitute a large group of zinc-dependent endopeptidases which have the capability of cleaving protein constituents of the extracellular matrix; MMPs may also activate or inactivate particular signaling molecules including adhesion molecules, receptors and growth factors (1, 2). MMP family members are broadly categorized into the following groups of enzymes: collagenases, stromelysins, gelatinases, and membrane-type metalloproteinases (3, 4). They normally exist in an inactive pro-form and require conversion to their active forms (5, 6). The activity of MMPs is also controlled by endogenous inhibitors, the tissue inhibitors of MMPs (TIMPs) (7, 8), and an endogenous stimulator, extracellular matrix metalloproteinase inducer (EMMPRIN) (8, 9). In addition, plasmin can activate MMPs to degrade a range of extracellular matrix molecules (10–12). Reactive oxygen species (ROS) also contribute to MMP activity (13, 14) by activating the preforms of MMPs, or inducing expression of their mRNA through signaling via NF-κB (5).

Activated MMPs are implicated in many processes such as cell survival, signaling, angiogenesis, inflammation, and cell motility (4, 15). They may directly injure brain cells by means of processing death molecules, disrupting myelin, and perpetuating neuroinflammation (4–6).

Among MMP members, the most important may be MMP−9. It is implicated in the remodeling and stabilization of dendritic spines, pre and post-synaptic receptor dynamics, consolidation of long term potentiation, synaptic pruning and myelin formation (16–18). MMP-9 is also involved in the sprouting, pathfinding and regeneration of axons (4, 19). MMP-9 is normally expressed in barely detectable level in the brain but after an injury, it is strongly detected in many cell types including endothelial cells and infiltrated neutrophils (20, 21). MMP-9 (Gelatinase B) is induced after injury through factors such as the c-fos and c-june, immediate early genes and by the cytokines, TNF-α and interleukin-1β (4, 6).

Though the evidence on the deleterious effects of MMPs in neurological diseases is substantial (5, 6, 20), the roles of MMPs in depression remain limited. Recent studies have indeed linked MMP-9 and the symptomatology of depression (22, 23). The aim of this paper is to provide an updated and comprehensive review regarding the role of MMP-9 in the pathology of depression with an emphasis on the probablility that MMP-9 expression could be a potential biomarker of depression.

### MMP-9, Neural Structure/Function and Depression

MMPs are important in the reaction of cells to their microenvironment. Both secreted and membrane-bound forms of MMPs are implicated in pericellular proteolysis (24). By the proteolytic degradation or remodeling of extracellular matrix proteins while simultaneously activating cell surface receptor ligands, MMPs affect the differentiation, survival, migration and proliferation of cells; axonal growth and pathfinding of axons are also controlled (25, 26). Thus, MMPs have important functions in wound healing and repair (5).

The extracellular matrix consists of three principal compartments in the CNS: the basement membrane that line blood vessels, perineuronal nets around certain population of neurons, and the interstitial matrix between neural cells (26). MMPs can determine the integrity of the basement membrane and thus the blood-brain barrier (BBB) via degradation of extracellular matrix and basement membrane components (7, 8). As a hallmark of brain trauma/stress injury, the disruption of the BBB is related to the increased permeability of damaged endothelial cells (27), facilitating the entry of inflammatory molecules into the brain. This increased neuroinflammation is thought to mediate, at least in part, the brain abnormalities as well as the cognitive decline in white and gray matter in individuals with bipolar disorder (28). By integrating human and animal data, Najjar et al. linked oxidative stress, endothelial nitric oxide synthase uncoupling, low endothelial nitric oxide levels, and neuroinflammation to putative BBB and neurovascular abnormalities in major depressive disorder (29).

The perineuronal nets around many neurons form a physical structure enwrapping the cell soma and proximal processes, particularly around parvalbumin-expressing GABAergic neurons. The perineuronal net can constitute a barrier to the formation of new synaptic contacts (30, 31). In postnatal development, hyaluronan/chondroitin sulfate proteoglycans (CSPG)-based extracellular matrix forms perineuronal nets that compartmentalize the neuronal surface, and restricts the surface mobility of integral membrane proteins, including glutamate α-amino-3-hydroxy-5-methyl-4-isoxazolepropionic acid receptors (32). Thus, perineuronal nets are involved in synaptic stabilization and limits synaptic plasticity (33). Very long-term memories such as fear conditioning are thought to be stored as hole patterns in the perineuronal net (34). Aberrant perineuronal signaling is proposed to induce CNS dysfunctions such as stroke, epilepsy and Alzheimer's disease (35).

Interestingly, some of the most effective treatments for mood disorders affect extracellular matrix molecules in perineuronal nets (32). In mice at postnatal 4–8 weeks, MMP-9 is inferred to impact cerebellar synaptic plasticity and perineuronal net remodeling, as MMP-9 activity is colocalized with synaptic markers and perineuronal nets (36, 37). MMP-9 decreases the integrity of perineuronal net around cortical neurons and this is associated with increased branching of input excitatory neurons while simultaneously lowering inhibitory input to these cortical neurons (38). Similarly, perineuronal net density around parvalbumin-expressing hippocampal interneurons is elevated in mice with social defeat-induced persistent stress disorder, which was abrogated by a 3-week antidepressant treatment (39). However, whether MMP-9 activity participates in the pathophysiology of depression through perineuronal net remodeling still needs more investigations.

The extension of multiple oligodendroglial branched processes toward axons is a significant event during the early stages of myelination; this prominent output of processes by oligodendrocytes likely requires remodeling of the extracellular matrix and participation of MMPs (40). Larsen et al. showed that an increase in MMP-12 and−9 in early postnatal development was beneficial to regulate myelinogenesis (41). As well, the involvement of MMPs in demyelination has been reported by several groups (42–44). Demyelination has also been reported in neuropsychiatric diseases such as depression and autism (45). If there is subsequent attempt at remyelination, the involvement of MMP-9 may be postulated.

**Figure 1:**
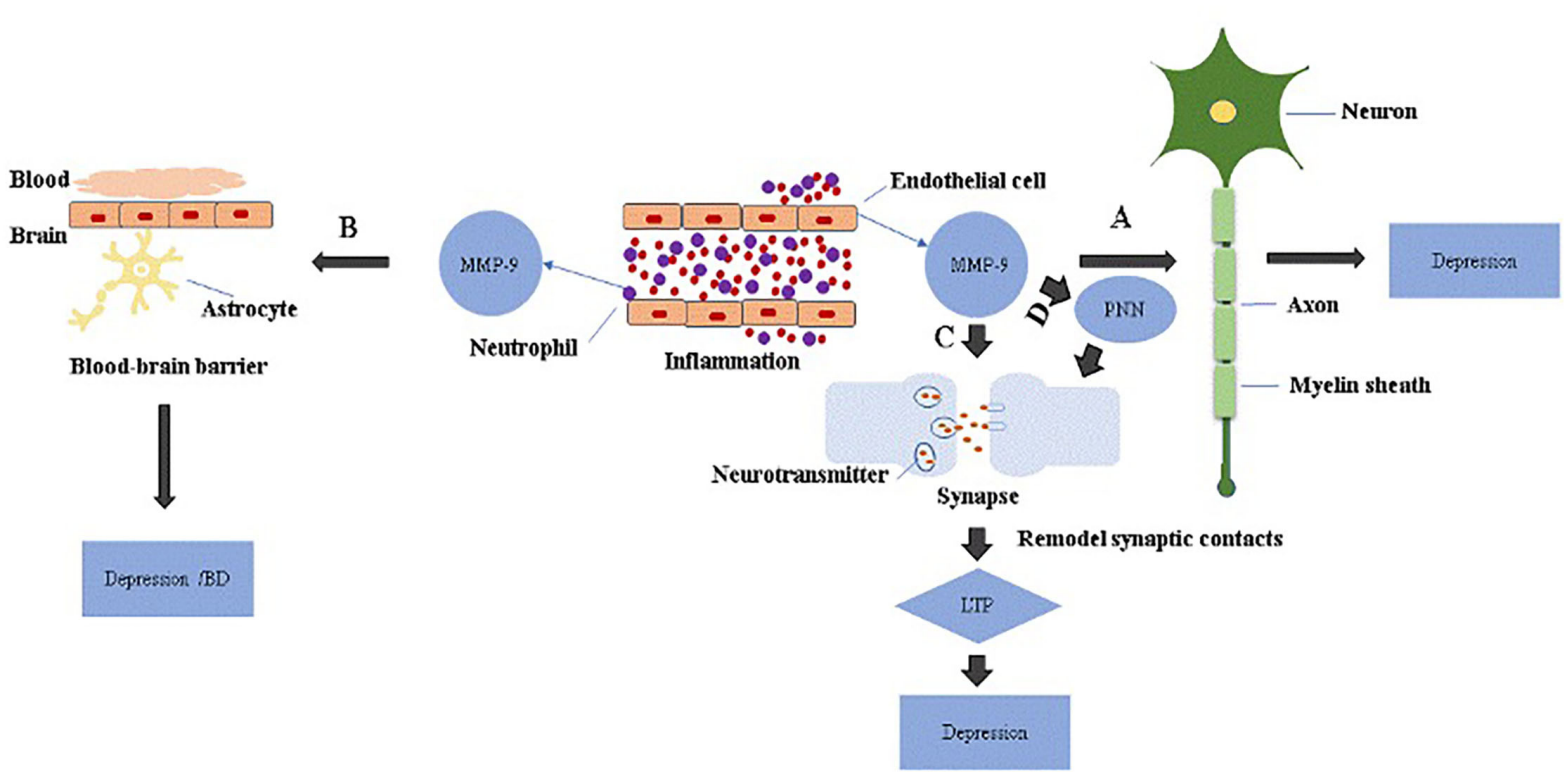
The role of MMP-9 in the pathology of depression. MMP-9 is elevated in endothelial cells and neutrophils during inflammation. **(A)** Excessive MMP-9 is thought to be involved in demyelination associated with depression. **(B)** MMP-9 disrupts BBB through tight junction proteins or basement membrane degradation, which increases neuroinflammation and may be linked to depression or bipolar disorders with cognitive decline. **(C)** Activated MMP-9 localizes in part to synapses and is involved in synaptic pruning essential for longterm potentiation (LTP), and attenuation of cortical synaptic LTP-like plasticity; collectively, these are thought to contribute to depression. **(D)** MMP-9 remodels perineuronal nets that participate in synaptic stabilization and limit synaptic plasticity. Depression may occur when perineuronal net signaling is aberrant.

Clinical studies report that cortical synaptic long-term potentiation (LTP)-like plasticity is attenuated in major depressive disorders patients, which recover after remission (46). The enduring nature of LTP has been attributed, in part, to long-term structural remodeling of synaptic contacts. For example, growth of new dendritic spines, increased density or formation of multi-synapse boutons, and enlargement of spine heads have all been associated with enduring LTP (47, 48). Activated MMP-9 partially localizes to synapses and has been proposed to modulate hippocampal synapses through integrin receptors; in this regard, blocking integrin function prevents an MMP-9-mediated potentiation of synaptic signal strength (48, 49). Moreover, extracellular conversion of proBDNF to active mBDNF is fundamental for late-phase LTP, and this appears to be mediated by extracellular proteases including plasmin/tissue plasminogen activator and/or MMP-9 (50). These observations suggest strongly that MMP-9-related LTP formation may be linked with depression (Figure 1).

### MMP-9 Gene Polymorphisms and Depression

MMP-9 gene functional polymorphisms may influence MMP-9 concentration, and MMP-9 activity highly depends on its expression level. The MMP-9 gene is located on chromosome 20q12.2–13.1, and it has three major polymorphisms in the untranslated, coding and promoter regions. Among these, the C-1562T (rs 3918242) polymorphism in the promoter region is of interest (51), because the promoter activity in driving gene expression is higher in the T-1562 allele than in the C-1562 allele (52). Galal et al. indicated that MMP-9 levels associated with CC genotype (C-1562T) polymorphism is significantly increased compared with CT genotype (51). Rybakowski et al. analyzed the functional-1562 C/T polymorphism genotype in a cohort of 416 patients with bipolar mood disorder and 558 healthy control subjects. T allele of the −1562 C/T polymorphism in the MMP-9 had a higher preponderance vs. the C allele in patients with bipolar mood disorder compared to healthy control subjects. Seventy five patients with bipolar disorder type II in a subgroup has much higher frequency of the T allele compared to the healthy group (53). In another study, Bobińska et al. examined 203 individuals suffering from depression and 99 healthy control individuals (54). The presence of the T-1702A polymorphism for MMP-9 elevates the risk of recurrent depression for the T allele and T/T genotype, whereas the A allele and A/A genotype decrease this recurrence. The C allele and C/C genotype of the C1562T MMP-9 polymorphism increase the risk of depression in midlife, while the T allele lowers this risk. In the case of a set of T/T-G/C genotypes of the MMP-9 TIMP-2G-418C and T-1702A polymorphisms, a higher incidence was also noted (54). These evidence support MMP-9 as a pathological mediator in depression. Further studies focusing on alterations of MMPs over the course of depression are warranted and required.

### MMP-9 as a Biomarker for Depression

Besides gene polymorphisms, alteration of MMP-9 levels has also been reported in depressive patients (Table 1). Yoshida et al. enrolled 69 patients with major depressive disorder and 78 age- and gender-matched healthy individuals. Mature brain-derived neurotrophic factor (BDNF) serum levels were significantly lower in patients with major depressive disorders than those in healthy individuals. In contrast, the serum levels of MMP-9 and proBDNF did not differ between patients and healthy subjects. Interestingly, these authors noted a positive correlation between the severity of depression and serum MMP-9 levels in patients with major depressive disorders. Increased MMP-9 expression was considered to be as a compensatory response to reduction of mature BDNF in patients with major depressive disorder (56).

**Table 1:** MMP-9 as a potential biomarker for depression—results from clinical studies.

**References**	**Study population**	**Study design**	**Main findings**
Domenici et al. (55)	728	245 patients with depression, 229 patients with schizophrenia, and 254 controls subjects; patients met DSM criteria; primary outcome: plasma levels of MMP-9	MMP-9 was higher in patients with depression vs. control group
Yoshida et al. (56)	147	69 patients with major depressive disorders, 78 control subjects; all patients met DSM-IV criteria for major depressive disorders and were outpatients; 65 patients were treated with antidepressants, 2 patients were treated with anxiolytics; primary outcome: serum levels of MMP-9	MMP-9 serum levels were associated with the severity of depression
Chiarani et al. (57)	40	20 patients with bipolar disorder, 20 control subjects; 50% patients were treated with haloperidol or chlorpromazine, and the others were treated with risperidone, olanzapine or clozapine. Primary outcome: MMP mRNA levels of blood cells using PCR	MMP-9 and MMP-2 expression in blood were not different between patients with bipolar disorder and control group.
Hufner et al. (58)	26	Participants were assessed during two separate research visits, one without and the other with ongoing mental stress which consisted of an average of 3 months preparation for a major university exam. Primary outcome: plasma levels of MMP-9	The interaction of acute physical and persistent mental stress led to a significant increase in plasma MMP-9.
Bobińska et al. (54)	242	142 patients with depression, 100 control subjects; patients were treated with antidepressants; primary outcome: MMP-9 gene expression of blood at the mRNA level	MMP-9 mRNA level was higher in patients with depression than in the control group.
Bobińska et al. (54)	234	139 patients with recurrent depression, 95 control subjects; patients were treated with antidepressants; primary outcome: MMP-2, MMP-9 and TIMP-2 gene expression of blood at the protein and mRNA level	MMP-9, MMP-2 and TIMP-2 expression was lower in depressive patients at both protein and mRNA levels than in the control group. Elevated expression of MMP-9, MMP-2 and TIMP-2 positively influences cognitive efficiency.
Chandrasekaran et al. (59)	25	A cross-sectional pilot study, 25 patients with bipolar disorder, primary outcome: serum levels of MMP-9	MMP-9 serum levels were higher in patients with a long history of suicidal thoughts compared to those without.

In another study, Domenici et al. reported that MMP-9 in serum was significantly higher in patients with major depressive disorders (*n* = 245) vs. controls (60). Rybakowski et al. performed a study on 54 in-patients with bipolar mood disorder and 29 control subjects. An increase of serum MMP-9 at the early stages of bipolar illness is found to accompany only the depressive episodes and not manic ones. Elevated levels of serum MMP-9 during depression in young patients may indicate that this phenomenon is a potential biochemical marker for the staging of bipolar disorder. The reason for the change in MMP-9 only at one stage of bipolar disorder is not known (61). Bobińska et al. examined a population comprised of 142 individuals suffering from depression and 100 control individuals. For all measured MMPs (MMP-9, MMP-2, MMP-7) and TIMP-2 in blood, increased gene expression was statistically more significant at the mRNA level in patients with depression as compared to control individuals (62). Domenici et al. conducted multiple analyte profiling of plasma samples from 245 depression patients and 254 controls. Increased MMP-9 levels, and to a lesser extent decreased MMP-2 levels, were documented in the depression group (55). In contrast, opposite observations have also been noted. Bobińska et al. reported decreased expression of transcripts and proteins of MMP-9, MMP-2 and TIMP-2 in blood in depression (63).

Some studies also reported no obvious correlation between MMP-9 level and depression. Platelet immunomodulatory and inflammatory properties are mediated through bioactive molecules mainly stored in cytoplasm and platelet granules. MMP-9 is found mainly in the cytosol of platelets. No group differences between major depressive disorders and controls were found for MMP-9 in platelets (58). Chiarani et al. recruited 20 patients with bipolar disorder and 20 control subjects that were matched for age and sex. They measured MMP levels in blood using real-time quantitative polymerase chain reaction. Pattern of MMP-9 expression has no difference between patients with bipolar disorder and control group (57). Large-scale multicenter studies are still necessary to analyse the role of MMP-9 as a contributor for depression.

Hemorrhagic transformation is exacerbated by thrombolytic therapy, which is a common complication of ischemic stroke. Jickling et al. proposed that early hemorrhagic transformation (<18 to 24 hours after stroke onset) is related to brain-derived MMP-2 and leukocyte-derived MMP-9 promotes BBB disruption and damage the neurovascular unit (64). It is proposed that the combination of tissue plasminogen activator along with an MMP-9 inhibitor can be beneficial in ischemic stroke (65). There are constitutively expressed MMPs that initiate the injury cascade early in the acute hypoxic/ischemic phase and inducible MMPs that perpetuate the damage over h and days (66). Thus, MMP-9 may be involved in the pathophysiologic process of stroke. Interestingly, there is emerging evidence of a bi-directional relationship between depression and cerebrovascular diseases, both of which are common conditions in older humans. In the first month after stroke, the frequency of post-stroke depression is highest, and remains high even several years later (67). Although MMP-9 may be involved in the pathophysiologic process of stroke and depression, whether it plays an important role in depression after stroke is uncertain. Furthermore, no evidence has been found to support the use of a particular biomarker for post-stroke depression (68).

### Stress and MMP-9 Activity

Stress is a risk factor for the development of psychopathologies characterized by deregulated social behaviors and cognitive dysfunction. Using a blister chamber wound model on human forearm skin exposed to UV-B, Yanga et al. found that depressive symptoms were reliably related to modulation of either TIMP or MMP expression. Moreover, activation of the sympathetic–adrenal medullary and hypothalamic–pituitary–adrenal axes can modulate levels of MMPs (69). In another depressive-like model, chronic mild stress decreased the concentration of the mature form of NGF and increased the active forms of MMP-2 and MMP-9 in the rat hypothalamus. Activated MMP-9 and MMP-2 cleaved the mature but not the pro-form of NGF into biologically inactive products (70).

Similarly, MMP-9-related gelatinase activity was elevated in the hippocampal CA1 of chronic restraint-stressed rats. Consistently, intra-CA1 administration of an MMP-9 inhibitor during stress exposure prevented the development of stress-induced deficits in social memory, social exploration and CA1-dependent cognition (71). However, in prenatal stress induced depressive-like rat model, gelatin zymography showed no significant change of MMP-9 and MMP-2 activity of both sexes (72). Further studies are necessary to determine the link between underlying mechanisms of stress and MMP-9.

### Antidepressants/Electro-Convulsive Therapy and MMP-9 Activity

Accumulated studies suggest that antidepressants may influence MMP-9 activity. For example, the antidepressant imipramine is reported to elevate the expression of MMPs to cleave perineuronal net proteins and affect inhibitory input to parvalbumin neurons in the hippocampus (37). Many antidepressants affect serotonergic neurotransmission and it is reported that stimulation of serotonin 5-HT7 receptor activated MMP-9 which cleaved the CD44 hyaluronan receptor on neurons leading to activation of the GTPase Cdc42; the authors proposed that this MMP-9-mediated pathway promoted synaptic pruning and impaired LTP, and caused reversal learning (73). Puscian et al. combined a study of neuronal plasticity in the central and basolateral amygdala with an automated assessment of motivation and learning in mice. They indicate that chronic treatment with fluoxetine in unstressed mice attenuated MMP-9-dependent plasticity in the central amygdala, while increasing perineuronal net-dependent plasticity in the basolateral amygdala (74). Alaiyed et al. reported that MMP-9 levels were elevated in prefrontal cortex of antidepressant-treated patients with major depressive disorders (38).

The clinical effects of lithium are well-understood, as it has been used in bipolar patients for over 60 years. Treatment of mesenchymal stem cells (MSCs) with lithium (2.5 mM for 1 day) selectively increased the protein/enzymatic and transcript levels of MMP-9. It was further demonstrated that lithium promoted migration of MSCs by up-regulation of MMP-9 through GSK-3β inhibition in rodents (75).

The antidepressant amitriptyline is reported in astrocglial cells to evoke glial-derived neurtotrophic factor production through a mechanism that is independent of monoamines, via activation of a pertussis toxin-sensitive Gi/o/MMP/fibroblast growth factor receptor (FGFR)/FRS2α/ERK cascade (76). Whether amitriptyline affects MMP expression or enzymatic activity remains to be identified. Another study showed that treatment with amitriptyline elevated zymographic MMP-9 activity without changing MMP-9 transcripts in C6 cells, and that MMP-3 was necessary to activate MMP-9 (77, 78).

Shibasaki et al. analyzed serum obtained from 21 patients with major depressive disorders and 40 healthy controls. Serum levels of TIMPs and MMPs were quantified by ELISA. These levels did not significantly differ from those of the control group before electro-convulsive therapy (ECT), but MMP-9 level was significantly reduced after ECT. Moreover, a significant positive correlation was observed between Hamilton Rating Scale for Depression (HAMD) scores and MMP-9 level in the serum (79). Benekareddy et al. demonstrated that chronic and acute ECT differentially regulated the transcript levels of TIMPs 1-4 and MMP-2/9 in rodents, and that ECT also increased activity of MMP-2/9 in the hippocampus. Chronic and acute pharmacological antidepressants, on the other hand, altered the expression of TIMPs without any observed impact on hippocampal MMP-2/9 activity or expression (80).

### MMP-9 Inhibitors and Depression

Based on it being an MMP-9 inhibitor and microglial activation blocker (81), the tetracycline antibiotic minocycline has been proposed for the treatment of depressive symptoms as well as negative symptoms in schizophrenia (82–84). It has been reported in rodents that minocycline reduced the effect of stress on working memory and neuronal activation, as well as microglial activation (37, 85). In the forced swimming test, minocycline produced antidepressant-like actions; subthreshold doses of both minocycline and desipramine in combination produced antidepressant-like actions (84, 86). In the testicular torsion/detorsion induced depression model and in the forced swimming test in rats, 160 mg/kg minocycline showed high antidepressant-like effect (84, 86). The nitric oxide/cGMP pathway was implicated in testicular torsion/detorsion-induced depressive-like behavior and minocycline had an antidepressant-like activity in this model (87). Repeated exposure to malathion leads to depressive-like behavior and minocycline significantly decreased immobility times in the tail suspension test and forced swimming test, associated with decreased hippocampal nitrite concentration (87). These investigations suggest an essential role for NO/cGMP pathway in antidepressant-like effect of minocycline in malathion-induced depressive-like behavior (88). Similarly, infusion of minocycline into the cerebral ventricle of learned helplessness rats induced antidepressant-like effects (89). Furthermore, in a rat model beginning 3 d before chronic unpredictable stress therapy and continuing through the behavioral testing period, chronic treatment with minocycline (120 mg/kg per day) prevented impairments of LTP induction and spatial memory (90). In another study, both chronic mild stressed rats or controls received twice intracerebroventricular injection of minocycline (160 μg) or vehicle. Minocycline was found to have neuroprotective effects through regulating energy metabolism and reducing oxidative damage in specific brain areas (91). In addition, chronic but not acute minocycline administration attenuated olfactory bulbectomized-induced depressive-like behavior (92, 93).

The above preclinical results are consistent with clinical studies. Soczynska et al. provided the rationale for organizing a randomized, controlled trial to examine the antidepressant properties of minocycline (94). Nettis et al. conducted a 4-week, placebo-controlled, randomized clinical trial of minocycline (200 mg/d) added to antidepressant therapy in 39 patients selected for elevated levels of serum C-reactive protein (CRP ≥ 1 mg/L); the authors found efficacy of add-on minocycline therapy in patients with major depressive disorder, but only in those with low grade inflammation defined as CRP ≥ 3 mg/L (95). In a 6-week, open-label study, 150 mg/day minocycline in combination with antidepressants (paroxetine, fluvoxamine, and sertraline) produced improvement in depression scores (96).

However, there are contradictory results concerning the antidepressant effects of minocycline. In C57BL/6 mice, minocycline (20–40 mg/kg, i.p.) did not cause anxiolytic - or antidepressant -like behavioral changes in contrast to mice treated with imipramine (20 mg/kg, depressive-like behavior) or diazepam (0.5 mg/kg, anxiety tests) (97). Furthermore, 266 patients were randomly assigned to receive celecoxib plus minocycline (*n* = 68), placebo plus minocycline (*n* = 66), placebo plus celecoxib (*n* = 66), or placebo plus placebo (*n* = 66). This double-blind, 12-week, randomized, placebo-controlled trial indicated that decreases in HAMD-17 was not different for patients treated with celecoxib or minocycline (98). Similarly, Krynicki et al. conducted a randomized double-blind, placebo-controlled trial of minocycline in 207 patients within 5 years of onset of schizophrenia, and found that minocycline did not affect any scores including depression and negative symptoms of schizophrenia (99).

## Concluding Remarks

MMP-9 plays important roles in BBB integrity, perineuronal net remodeling, myelinogenesis, and synaptic physiology. Clinical studies suggest that MMP-9 gene polymorphisms are related to depressive symptoms, and altered MMP-9 levels are observed in depressed patients and in depressive-like animal models (51–54). It is to be noted that the literature on MMP-9 is more established for bipolar disorder rather than depression. Nonetheless, accumulated studies indicate that serum MMP-9 may be a novel therapeutic target and biomarker for depression (55–57, 60–63), although a cautionary note is that blood level of MMP-9 may not directly correlate with brain MMP-9 content. MMP-9 appears to be a target for classical antidepressant treatments and MMP-9 inhibitors possess potential therapeutic effects for depression.

In summary, we suggest MMP-9 to be an important factor in depression, not only as a therapeutic target but also as a biomarker in the condition.

## Author Contributions

All authors contributed to the writing or revising of the manuscript. All authors contributed to the article and approved the submitted version.

## Funding

The authors acknowledge operating grant support from the National Natural Science Foundation of China (Grant Nos. 82071331, 81870942, and 81520108011), National Key Research and Development Program of China (Grant No. 2018YFC1312200) (MX), and from the Canadian Institutes of Health Sciences (VY).

## Conflict of Interest

The authors declare that the research was conducted in the absence of any commercial or financial relationships that could be construed as a potential conflict of interest.

## Publisher's Note

All claims expressed in this article are solely those of the authors and do not necessarily represent those of their affiliated organizations, or those of the publisher, the editors and the reviewers. Any product that may be evaluated in this article, or claim that may be made by its manufacturer, is not guaranteed or endorsed by the publisher.

## Abbreviations

CNS: central nervous system
MMP−9: Matrix metalloproteinase-9
PNNs: perineuronal nets
MMPs: Matrix metalloproteinases
TIMPs: tissue inhibitors of metalloproteinases
EMMPRIN: extracellular matrix metalloproteinase inducer
ROS: Reactive oxygen species
LTP: long term potentiation
BM: basement membrane
BDNF: brain derived neurotrophic factor
BBB: Brain-blood barrier
TJPs: tight junction proteins
BD: bipolar disorder
CSPG: chondroitin sulfate proteoglycans
PCR: polymerase chain reaction
NGF: Nerve growth factor
MSCs: mesenchymal stem cells
FGFR: fibroblast growth factor receptors
HAMD: Hamilton Rating Scale for Depression.
